# A case of tumor-related hemobilia successfully treated with radiofrequency ablation diagnosed by peroral cholangioscopy

**DOI:** 10.1055/a-2719-3047

**Published:** 2025-11-14

**Authors:** Takahiro Urata, Shun Kawahara, Hideki Kitada

**Affiliations:** 1 38346 Department of Gastroenterology, Japanese Red Cross Kumamoto Hospital, Kumamoto, Japan

Radiofrequency ablation (RFA) is a treatment that uses high frequency electrical current to induce coagulative necrosis of tumors. In the biliary tract, RFA has been reported as a palliative option to reduce tumor burden and maintain ductal patency, often combined with stenting; however, strong evidence supporting its efficacy remains limited 1 2 3 . Hemobilia, especially when tumor-related, presents significant diagnostic and therapeutic challenges and can be life-threatening. We report a case in which the bleeding source was identified via peroral cholangioscopy (POCS), and hemostasis was successfully achieved using RFA.

A 72-year-old man presented with persistent epigastric pain. Laboratory tests revealed elevated total bilirubin (4.2 mg/dL), AST (518 U/L), ALT (304 U/L), and ALP (228 U/L). Contrast-enhanced computed tomography (CT) showed a hypovascular tumor in segment 4 ( Fig. 1 ) and a hyperdense area within the bile duct, suggestive of intraductal blood. Endoscopic retrograde cholangiopancreatography (ERCP) revealed a radiolucent filling defect, which was removed using a balloon catheter and identified as a hematoma ( Fig. 2 **a, b** ). Endoscopic nasobiliary drainage was performed to relieve jaundice and inflammation. Once jaundice improved, POCS revealed an exposed tumor in the left intrahepatic bile duct ( Fig. 3 ), with ongoing tumor-related bleeding observed ( Video 1 ). Biopsy confirmed adenocarcinoma.

**Fig. 1:**
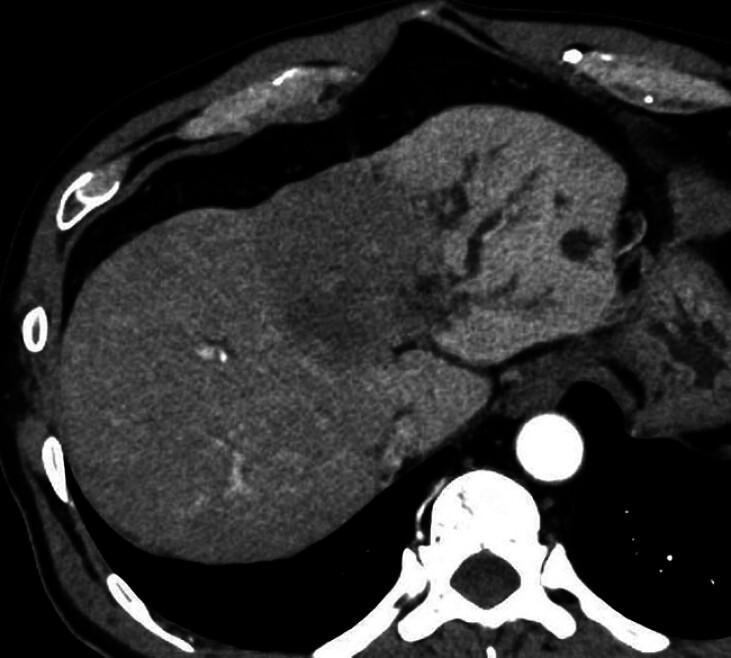
Contrast-enhanced abdominal computed tomography showed a hypovascular tumor in hepatic segment 4.

**Fig. 2:**
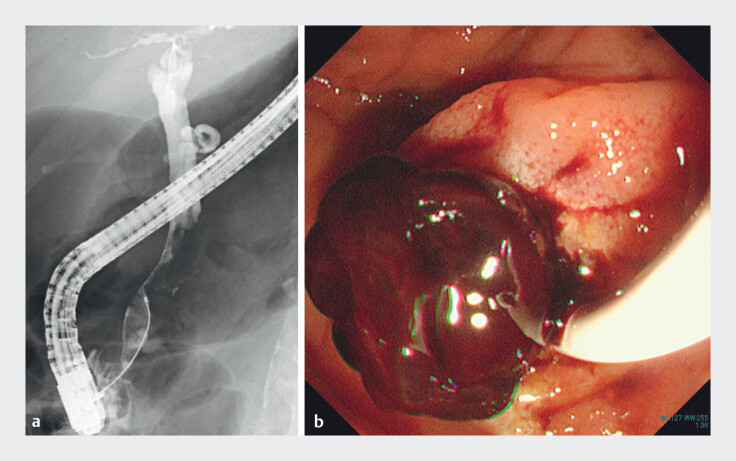
**a** Endoscopic retrograde cholangiopancreatography showed a radiolucent area (arrowhead) in the bile duct. **b** The lesion was identified as a blood clot after removal with a balloon catheter.

**Fig. 3:**
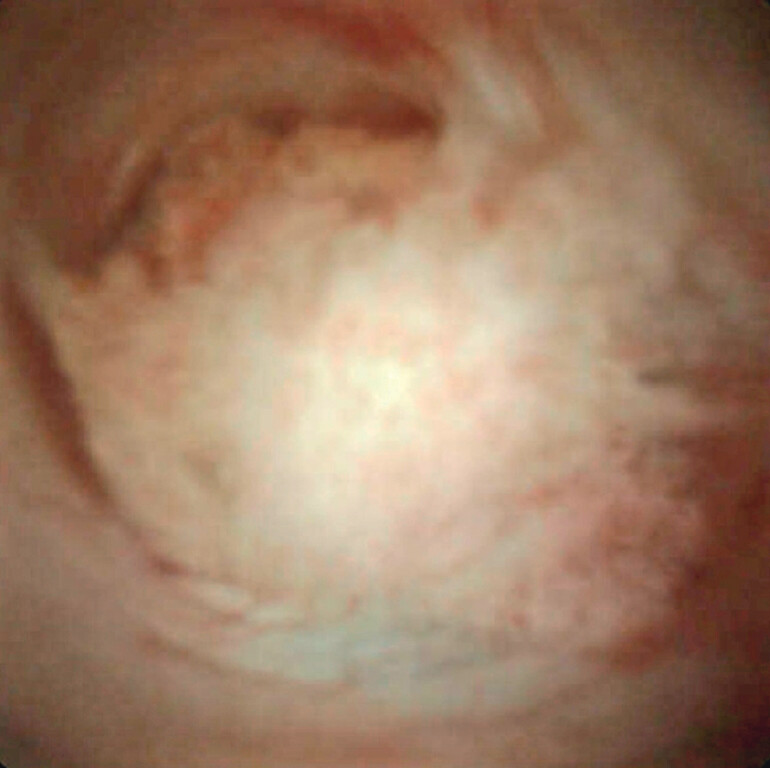
Peroral cholangioscopy (POCS) revealed that the tumor was exposed to the lumen of the left intrahepatic bile duct.

Peroral cholangioscopy revealed tumor-related hemobilia, and hemostasis was achieved with repeated sessions of intraductal radiofrequency ablation.Video 1

RFA was performed using a Habib EndoHPB catheter (Boston Scientific, Marlborough, Massachusetts, USA). The first session was applied at 7 W for 90 seconds, then 10 W for 90 seconds. Follow-up POCS one week later showed partial tumor ablation but mild persistent bleeding. A second RFA session at 10 W for 90 seconds, repeated twice, achieved hemostasis ( Fig. 4 , Video 1 ). The patient’s condition improved without anemia progression, and chemotherapy with gemcitabine and S-1 was initiated. No tumor-related bleeding recurred during seven months of follow-up.

**Fig. 4:**
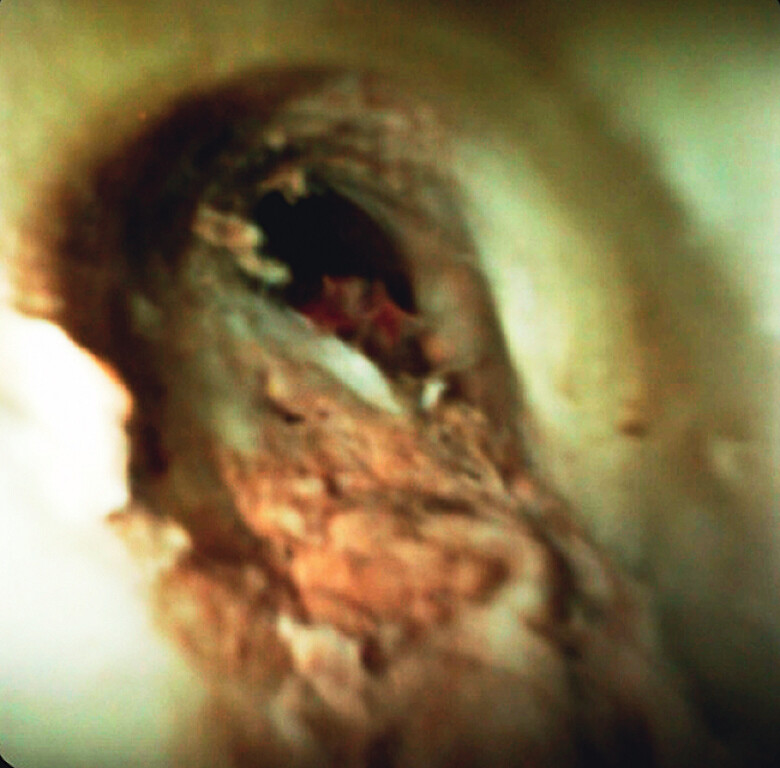
POCS confirmed successful tumor ablation and hemostasis.

Endoscopy_UCTN_Code_TTT_1AR_2AF

## Notes

Endoscopy E-Videos https://eref.thieme.de/e-videos
*E-Videos* is an open access online section of the journal *Endoscopy* , reporting on interesting cases and new techniques in gastroenterological endoscopy. All papers include a high-quality video and are published with a Creative Commons CC-BY license. Endoscopy E-Videos qualify for HINARI discounts and waivers and eligibility is automatically checked during the submission process. We grant 100% waivers to articles whose corresponding authors are based in Group A countries and 50% waivers to those who are based in Group B countries as classified by Research4Life (see: https://www.research4life.org/access/eligibility/ ). This section has its own submission website at https://mc.manuscriptcentral.com/e-videos .
